# Draft Genome Sequence of *Neofusicoccum parvum* Isolate UCR-NP2, a Fungal Vascular Pathogen Associated with Grapevine Cankers

**DOI:** 10.1128/genomeA.00339-13

**Published:** 2013-06-13

**Authors:** Barbara Blanco-Ulate, Philippe Rolshausen, Dario Cantu

**Affiliations:** Department of Viticulture and Enology, University of California—Davis, Davis, California, USAa; Department of Plant Sciences, University of California—Davis, Davis, California, USAb; Department of Botany & Plant Sciences, University of California—Riverside, Riverside, California, USAc

## Abstract

*Neofusicoccum parvum*, a member of the *Botryosphaeriaceae* family, is a vascular pathogen that causes severe decline and dieback symptoms in grapevines worldwide. The draft genome of the grapevine isolate *N. parvum* UCR-NP2 provides a first glimpse into the complex set of putative virulence factors that this pathogen may use to rapidly colonize plants.

## GENOME ANNOUNCEMENT

Several species in the *Botryosphaeriaceae* family, including *Neofusicoccum parvum* (Pennycook & Samuels) Crous, Slippers, and Phillips (teleomorph *Botryosphaeria parva*), are cosmopolitan opportunistic pathogens of grapevines and other economically important perennial plants (1). *N. parvum* penetrates grapevines through pruning wounds and colonizes the host tissues, causing shoot dieback, cane bleaching, bud necrosis, and graft failure (2, 3). Wedge-shaped necrosis in the vascular tissues of spurs, cordons, and trunks are typical symptoms of botryosphaeria cankers (2). Disease symptoms suggest the involvement of cell wall-degrading proteins and phytotoxins in the breakdown of the plant tissues and induction of cell death, respectively (2, 4). Progress in understanding the mechanisms underlying *N. parvum* pathogenicity have been hindered by the lack of genome sequence information, the variability in virulence to grapevines among the isolates, and the difficulty of distinguishing *N. parvum* disease symptoms from those caused by other vascular fungal pathogens (3, 5, 6).

*N. parvum* UCR-NP2 was obtained from the margin of a grapevine (*Vitis vinifera* cv. “Zinfandel”) wood canker collected in Riverside County (California) in 2011. Isolation and species identification were carried out as described previously (1). DNA was extracted using a modified cetyltrimethylammonium bromide (CTAB) method (7) and sequenced using the Illumina HiSeq 2000 platform to a median depth of 113× to guarantee sequencing accuracy at the nucleotide level. Assembly was performed with CLC Genomics Workbench v6.0 with the parameters optimized to achieve the best gene space assembly completeness estimated with Core Eukaryotic Genes Mapping Approach (CEGMA) (8). Sixty-three million paired-end reads were assembled into 1,877 contigs, which were further organized within 1,287 scaffolds (N_50_, 83 kb; L_50_, 149; gaps, 69 kb; G+C content, 56.7%) with a total sequence of 42.5 Mb, a genome size similar to those of other plant-pathogenic ascomycetes (9, 10). The UCR-NP2 gene space was estimated to be >98% complete by mapping 248 low-copy core eukaryotic genes (CEGs) conserved across higher eukaryotes to the scaffolds (8).

The gene structures of the CEGs identified in the UCR-NP2 genome were used to train Augustus (11) for *ab initio* gene discovery on scaffolds that were masked for repeats using RepeatMasker (http://repeatmasker.org). Augustus predicted 10,470 complete protein-coding genes, from which 96% are homologous to genes in the NCBI collection of ascomycete proteins, and 82% were annotated using BLAST similarity searches against the complete GenBank nr database (BLASTp, E value < 1e^-3^). Among the 1,097 proteins identified as potentially secreted (SignalP v4.0 [12]), we detected a set of enzymes that might function during the colonization of host tissues, which include 163 glycoside hydrolases, 22 polysaccharide lyases, and 8 cutinases annotated based on homology with proteins in the CAZy database (13). We also found 4 lignin peroxidases and 212 cytochrome P450 monooxygenases that might be involved in lignin degradation (14, 15). This remarkable expansion of P450s in UCR-NP2 is comparable to that found in other genomes of wood-decaying fungi, including *Eutypa lata* (205 P450s [16]), *Phanerochaete carnosa* (266 P450s [17]), and *Postia placenta* (236 P450s [18]).

### Nucleotide sequence accession numbers.

This Whole-Genome Shotgun project has been deposited at DDBJ/EMBL/GenBank under the accession no. AORE00000000. The version described in this paper is the first version, accession no. AORE01000000.
